# Supplementary material for: Qki regulates myelinogenesis through Srebp2-dependent cholesterol biosynthesis
Source: eLife. 2021 May 4;10:e60467. doi: 10.7554/eLife.60467 (PMC8139834; doi:10.7554/eLife.60467)
Supplement: Supplementary file 1. [file elife-60467-supp1.docx]

**Supplementary File 1.**

| **Primer Name** | **Primer sequence** | **Comments** |
| --- | --- | --- |
| *Hmgcs1*-Forward | TGGAAGCCTTTGGGGACG | RT-qPCR |
| *Hmgcs1*-Reverse | GGTGAGTACTGTGCCAGGAC | RT-qPCR |
| *Hmgcr*-Forward | CCAAGAGAGAAAAGTTGAGG | RT-qPCR |
| *Hmgcr*-Reverse | TTGCACCTTTCTCTGCATTC | RT-qPCR |
| *Mvk*-Forward | GGTGTGGTCGGAACTTCCC | RT-qPCR |
| *Mvk*-Reverse | CCTTGAGCGGGTTGGAGAC | RT-qPCR |
| *Pmvk*-Forward | AGGAGTATGCTCGGGAGCATG | RT-qPCR |
| *Pmvk*-Reverse | TGTGTCACTCACCAGCCAGATAG | RT-qPCR |
| *Mvd*-Forward | CAGCCAATGGAGACAAGTTCC | RT-qPCR |
| *Mvd*-Reverse | GTCCTGGTCCGACCTGAGTG | RT-qPCR |
| *Idi1*-Forward | ACCAGCCATCTTGATGAAAAACA | RT-qPCR |
| *Idi1*-Reverse | CAGCAACTATTGGTGAAACAACC | RT-qPCR |
| *Fdps*-Forward | GGAGGTCCTAGAGTACAATGCC | RT-qPCR |
| *Fdps*-Reverse | AAGCCTGGAGCAGTTCTACAC | RT-qPCR |
| *Fdft1*-Forward | GTGCTATTCCACAGGTAATG | RT-qPCR |
| *Fdft1*-Reverse | GATCCGGTGATAAATCTCTTC | RT-qPCR |
| *Sqle*-Forward | ATAAGAAATGCGGGGATGTCAC | RT-qPCR |
| *Sqle*-Reverse | ATATCCGAGAAGGCAGCGAAC | RT-qPCR |
| *Lss*-Forward | GGCTCCTGGGAGGGCTCCTG | RT-qPCR |
| *Lss*-Reverse | CTGCACAAGCAGCCCCATCTTGG | RT-qPCR |
| *Dhcr24*-Forward | CTCTGGGTGCGAGTGAAGG | RT-qPCR |
| *Dhcr24*-Reverse | TTCCCGGACCTGTTTCTGGAT | RT-qPCR |
| *Cyp51*-Forward | GACAGGAGGCAACTTGCTTTC | RT-qPCR |
| *Cyp51*-Reverse | GTGGACTTTTCGCTCCAGC | RT-qPCR |
| *Tm7sf2*-Forward | CTCATCTGCCTCCTTAAGGTTATTGG | RT-qPCR |
| *Tm7sf2*-Reverse | GGGATGGTCTCAAGACCAGCCAC | RT-qPCR |
| *Msmo1*-Forward | ATCACGAGTTTCAGGCTCCAT | RT-qPCR |
| *Msmo1*-Reverse | TTCAGCGGGTTGAGAGGAAT | RT-qPCR |
| *Nsdhl-*Forward | CAGGAGAGAGCAGTACTGGATG | RT-qPCR |
| *Nsdhl-*Reverse | CAGGTTTTCCCCATTTCCAATC | RT-qPCR |
| *Hsd17b7-*Forward | GAATTTCAACCAGAAGGGTCTG | RT-qPCR |
| *Hsd17b7-*Reverse | ACAAAAAAGCGAAGGAGCCAC | RT-qPCR |
| *Actb-*Forward | CCACCATGTACCCAGGCATT | RT-qPCR |
| *Actb-*Reverse | CCGATCCACACAGAGTACTT | RT-qPCR |
| C-*Hmgcs1*-Forward | GGTCGGTGGCTATAAAGCTG | ChIP-qPCR |
| C-*Hmgcs1*-Reverse | CGGGACACTCACCCAAAG | ChIP-qPCR |
| C-*Hmgcr*-Forward | GCTCGGAGACCAATAGGA | ChIP-qPCR |
| C-*Hmgcr*-Reverse | CCGCCAATAAGGAAGGAT | ChIP-qPCR |
| C-NC-Forward | ATGCCTAACTTCCAGTTCCAGG | ChIP-qPCR |
| C-NC-Reverse | AGCTTAGAGCAGAAAGCTGGT | ChIP-qPCR |
